# Increasing research capacity in an academically isolated mental health trust

**DOI:** 10.1192/bjb.2025.10180

**Published:** 2026-08

**Authors:** Adrian J. Hayes, Alicia Long, Carinna Vickers

**Affiliations:** 1 Consultant Medical Psychotherapist, Specialist Psychological Interventions, Somerset NHS Foundation Trust, Yeovil, UK; 2 Clinical Trials Officer, Department of Clinical Research, Somerset NHS Foundation Trust, Yeovil, UK; 3 Registered Nurse, Team Lead Research Nurse, Department of Clinical Research, Somerset NHS Foundation Trust, Yeovil, UK

**Keywords:** Patients and service users, mental health services, general adult psychiatry, evidence-based mental health, education and training

## Abstract

**Aims and method:**

Involvement in clinical mental health research can be a challenge for services isolated from academic institutions, limiting opportunities for patients to receive innovative interventions and for clinicians to explore interest in research. We aimed to increase mental health research capacity in Somerset NHS Foundation Trust via a range of initiatives from collaboration between a senior clinician and research and development colleagues.

**Results:**

Over the course of the project, the number of participants recruited to National Institute for Health and Care Research-adopted mental health and dementia research projects quadrupled over a 2-year period, from 57 to 232, and the number of projects hosted rose from 9 to 23. A total of 165 clinicians signed up to receive information about ongoing studies.

**Clinical implications:**

We found considerable appetite for becoming involved in research among mental health clinicians, and were able to provide opportunities for research experience as well as access to innovative studies for local patients.

Research plays a fundamental role in healthcare, in the development of new treatments and services, improving outcomes and driving innovation.^1^ There can be a disconnect between academics and clinicians in the translation of research findings into current clinical practice and access to interventions. There is a decline in the clinical academic workforce,^2^ and research can be pushed out of clinician job plans in favour of service delivery to meet increased service demands and enhance productivity, particularly when budgets are tight and staffing levels are low. Access to involvement in clinical research studies can be limited for health services that are not connected to academic institutions.^3^ Mental health research is underfunded in comparison with other health conditions, despite a large burden,^4^ and is failing to attract clinicians into academic posts.^5^ Inequalities in access to mental health research have also been identified, particularly for deprived areas and under-served communities, with Mental Health Research Groups representing one recent mechanism to address this.^6^

The Research Delivery Network (RDN) was set up in 2024 to ‘attract, optimise and deliver’ research projects across England, and replaced the Clinical Research Network, which had a similar purpose.^7^ Twelve regional centres coordinate and support research across geographical areas, including the National Health Service (NHS). The network provides a mechanism for research and development (R&D) departments in each NHS Trust to host research projects that align with local and national priorities. Studies registered with the RDN Portfolio, usually national studies with government funding, are prioritised and funded for delivery by R&D departments based on levels of activity. These departments usually have a delivery team with staffing, including research clinicians, but who may not be working in frontline clinical services but rather focusing on research delivery.

The South West Peninsula Research Delivery Network (formerly Clinical Research Network) funds a number of research associate awards for health and social care clinicians from Cornwall, Devon and Somerset to develop their research capacity and skills.^8^ Support is available for development of skills, research experience and mentoring to encourage involvement in the delivery of portfolio studies, along with support for developing research careers alongside clinical practice.

This paper is a description and evaluation of the work completed as part of a funded research associate post at Somerset NHS Foundation Trust. A.J.H. is a consultant medical psychotherapist funded as research associate (1.6 sessions per week for 2 years), who teamed up with A.L., a clinical trials officer in the local R&D department. Together they have implemented a range of initiatives designed to increase research capacity for the Trust’s mental health services, focusing on delivery of RDN Portfolio research studies.

## Method

### Setting

Somerset Foundation NHS Trust is located in a county with no university or teaching hospital, and no formal links with academic institutions in mental health research. It serves a rural geographical area with four centres, at Bridgwater, Wells, Yeovil and the county town of Taunton, along with a number of smaller outposts. In 2023, the Trust combined mental health services with two general health trusts, each with a district general hospital and accompanying community services serving around 340 000 people. In terms of training, it hosts medical residents from the Severn Deanery, clinical psychology trainees from the Universities of Bath and Exeter and nursing students from the Universities of the West of England and Plymouth.

### Initiatives

#### Targeted staff groups

We identified groups of staff who were required and/or motivated to gain research experience. Assistant psychologists and clinical associate psychologists are often working to gain a place on clinical psychology doctorate courses for which research experience is an essential factor. In discussion with operational managers and clinical supervisors, these staff members were able to ring-fence at least 1 hour per week for research in their job plans. Postgraduate doctors in training are obliged to meet portfolio competencies in research, audit and quality improvement during core and advanced training. Advanced clinical practitioners also have research as one of four ‘pillars’ in their role, and their involvement in portfolio studies was supported by the Trust associate director of patient care. We made efforts to ensure that all of these groups were using the time available to them to contribute to portfolio studies. Finally, we were successful in securing small amounts of funding to recruit research champions in psychology, pharmacy and social work, with plans to extend this to further staff groups. These clinicians encouraged their interdisciplinary colleagues to become involved in studies, sign up to the research network mailing list and highlight patients eligible for portfolio studies.

Clinical psychologists are trained as researcher clinicians in order that they can carry out research during their routine clinical jobs (although this rarely happens in practice).^9^ We gained agreement to include protected research time in clinical psychology job plans in advertisements for posts that had been difficult to recruit, focusing on in-patient psychology roles. We also encouraged senior clinicians (including non-psychologists) to offer supervision to trainee clinical psychologists conducting their mandatory research and quality improvement projects.

#### Research experience

We focused on clinicians gaining experience of research that was experiential and patient-facing, to illustrate that research could be a dynamic and clinically relevant practice. We offered a number of levels for involvement, and met with individual clinicians to explore their interests and needs in order to provide the optimal experience. Most simply, we encouraged clinicians to refer patients to us who were eligible for inclusion in portfolio studies. We supported them to look through both their own and team case-loads, to advertise studies among colleagues and to listen out for new eligible patients in referrals meetings.

For further involvement in portfolio studies, we required clinicians to complete the NIHR Good Clinical Practice e-learning module. The certificate for this course, along with a completed research curriculum vitae, were necessary for listing on the delegation log for an individual portfolio study. Once registered, clinicians could be trained to complete research tasks such as taking informed consent and completing outcome measures or clinical questionnaires. Once they had some experience, we encouraged them to take on more responsibility in formal roles such as associate principal investigator, co-investigator and principal investigator. We particularly encouraged our resident doctors in advanced psychiatry training to take on the associate principal investigator role, which offers a nationally recognised research experience via NIHR.

#### New studies

New portfolio studies often require that the principal investigator be a medical consultant, particularly for drug trials. Previous practice was that, when an upcoming study was being considered, the R&D team would email large groups of consultants asking whether anyone was interested in taking on the role of principal investigator. There was little uptake, because clinicians are very busy and this seemed an additional task with limited reward.

We altered this process to contact targeted groups of senior staff in relevant clinical areas, to involve them in the decision about whether to host a new study. We found greater interest in contributing to discussion about the project’s relevance, availability of the patient groups under study and what practical issues there may be in taking part. Clinicians were then invested in the project, and it was easier to recruit a principal investigator, with occasional competition for the role resulting in more co-investigators.

#### Service evaluations

We included support for service evaluations as part of the work in increasing research capacity. Service evaluations represent a good way for clinicians to conduct small-scale projects evaluating their practice or even new initiatives, if introduced for clinical reasons on a local basis. We discovered that there were many such innovations in Somerset, but few incorporated evaluation and they were rarely written up. A.J.H. hosted discussions for clinicians to develop evaluations, report these to the Directorate of Mental Health and Learning Disability and encourage them to disseminate their findings.

#### Monthly clinical research network meetings

We set up a monthly online meeting for clinicians across the Trust to hear about new and upcoming studies in mental health, learning disability and dementia. Teams were informed about the meetings and were encouraged to make contact to receive regular invitations, and were encouraged to pass on details to interested colleagues. At this meeting we updated attendees on current RDN Portfolio studies, emphasising participant eligibility to support clinicians in making contact with us if they worked with eligible patients. We brought new projects found on the NIHR Open Data Platform to discuss whether clinicians were interested in the initiatives under study, and whether they thought we would have eligible patients in the Trust. We arranged presentations from clinicians who were conducting research in the south-west region and also advertised relevant training courses, funding opportunities and local conferences. Following meetings, a comprehensive email was sent summarising the content and including a summary of ongoing portfolio projects. This created a local research community across disciplines and services, allowing for cross-pollination of ideas and development.

#### Lived experience pilot

We took part in a regional pilot project employing a lived experience research assistant (LXRA) who had previously been with mental health services and later worked as a peer support worker in the Trust. Our LXRA did not recruit directly for the studies, but raised awareness of them among staff and patient groups across the Trust and in the voluntary sector.

### Data

On completion of the 2-year project, we reviewed data on the number of mental health portfolio projects opened in the Trust, and the number of total participants recruited across all mental health portfolio projects during the period 2022–2024.

## Results

In 2022 the Trust was taking part in nine portfolio projects, several of which were long-term studies that had been in progress for some years. There were four questionnaire studies or surveys, three observational studies and two interventional studies. One band 5 clinical trials officer was delivering all these studies, supervised by a research nurse from the R&D department of the Trust. Six clinicians were named as principal investigators across these studies, and a further eight had been involved in helping set up or run the studies.

**Fig. 1:**
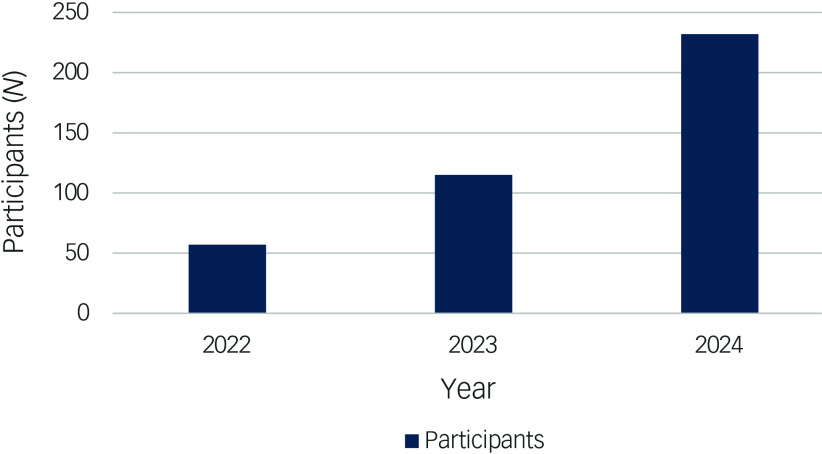
Recruitment of participants to portfolio studies between 2022 and 2024.

**Fig. 2:**
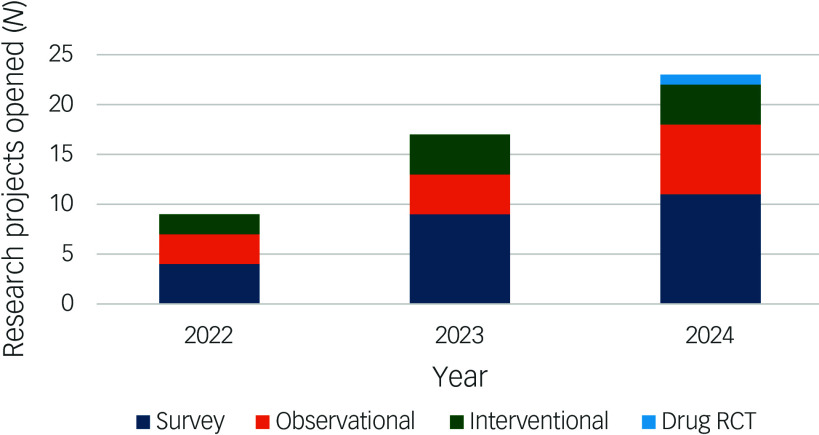
Number of portfolio studies opened between 2022 and 2024. RCT, randomised controlled trial.

The number of participants recruited to portfolio studies (Fig. 1) more than doubled in each year from 2022 to 2024, quadrupling overall from 57 to 232. The number of projects opened in the Trust increased from 9 to 23 in the 2-year period (Fig. 2), with an increase in the complexity of projects hosted (more interventional studies and drug trials). This increase allowed the Trust to take on a new part-time clinical trials officer. One assistant psychologist working clinically in the Trust also negotiated working 1 day per week with R&D in the delivery of portfolio studies. Fourteen clinicians were registered as new principal investigators, with a further 26 individual listed on delegation logs and formally working on projects.

A total of 165 clinicians signed up to the monthly clinical research network meetings and associated mailing list over the 2-year period.

Two in-patient clinical psychology posts were advertised with protected research time, neither of which had recruited for over 2 years. One achieved successful recruitment, with the research time mentioned as an incentive by the post-holder.

We registered 51 separate service evaluations that had either been completed or begun over the 2 years. Only two had been written up as journal articles, and the vast majority had not been presented outside of the Trust.

## Discussion

Our analysis of data showed that research capacity increased markedly over the 2 years of this work, as measured by the numbers of new projects opened and participants recruited. While we cannot show a causal link, we believe that the clinical research associate role and R&D collaboration were instrumental in driving this increase.

Collaborative working allowed front-line clinicians the opportunity to become involved in national research projects. Involvement was immersive and patient-facing, which appeared to counteract previously held attitudes that research was dry or bureaucratic while demonstrating relevance to their clinical expertise. Clinicians in training were able to meet research competencies and develop involvement in formal roles in more complex studies. More patients were able to become involved in clinical research locally and we were able to meet recruitment targets, potentially allowing for site selection in future competitive studies.

We believe that research can be an engaging addition to clinicians’ roles, providing variety of experience, additional choices to offer patients and an opportunity to be at the cutting edge of innovation. Where burnout is high and staff retention low, research may be a way of enthusing the workforce and retaining joy at work. It may also be an effective mechanism for boosting recruitment to underfilled posts. We would like to explore this further in a more focused evaluation.

Informal feedback has been that research opportunities can be of interest to clinicians, and those who have been involved in projects have had a positive experience. One clinical psychologist wrote:

‘My involvement in research has been a tonic. It has been like an intellectual holiday; an absorbing project to engage with, linked to but also outside of the constant pressure of NHS working. It has reignited my curiosity and passion for mental health and reminded me why I chose my profession. I have had the chance to dust-off old skills and learn some new ones and to engage with like-minded colleagues.’

Our work may have implications for other similar sites that do not have good access to academic institutions and local research centres. We believe that it may also be of interest for sites that do have such links, because there can be a disconnect between academic research and the clinical services at which academic centres are based. Research at high-level institutions can be seen as elite and suitable only for large-scale projects with long timescales. In our experience, clinicians becoming involved with major projects are rarely able to gain a short, interesting experience in the practical aspect of engaging with patients to collect data in the way we have provided.

We believe that there is an important role for ‘research delivery clinicians’ in NHS medical services, which we would distinguish from ‘academic clinicians’. While academic clinicians are applying for grants, publishing papers and finding ways of testing their own ideas and innovations in large-scale studies, research delivery medics would be those supporting large studies on a local basis, ensuring that they are made available to their patients and mobilising other clinicians to become involved. The work offers the opportunity to become involved in the more dynamic aspects of research delivery, and avoids some of the more bureaucratic elements such as grant-seeking, ethical approval and data analysis. Advanced technical research or academic writing skills are not necessary for this role, but more valuable are communication, networking and maintaining motivation among clinical colleagues.

There is currently little prestige attached to being a research delivery medic in terms of academic status or direct funding. However, we have shown that this role can greatly increase research capacity in local NHS services. This provides more funding to trusts via R&D departments, and we believe that it can increase job satisfaction and collaborative multidisciplinary working. We believe that, if the role were seen as valuable by leaders and facilitators in their organisation and in the research community, further research opportunities could be made available, thus ensuring that our patients have access to the newest treatments and reducing inequalities.

## About the authors

**Adrian J. Hayes**, MRCPsych, MBChB, PhD, is a consultant medical psychotherapist, **Alicia Long** is a clinical trials officer and **Carinna Vickers**, a registered nurse, is a team lead research nurse, all at Somerset NHS Foundation Trust, Yeovil, UK.
